# *Saccharomyces cerevisiae* Promoter Engineering before and during the Synthetic Biology Era

**DOI:** 10.3390/biology10060504

**Published:** 2021-06-06

**Authors:** Xiaofan Feng, Mario Andrea Marchisio

**Affiliations:** School of Pharmaceutical Science and Technology, Tianjin University, 92 Weijin Road, Tianjin 300072, China; fengxf95@tju.edu.cn

**Keywords:** promoter, synthetic biology, transcription factors, gene expression, *Saccharomyces cerevisiae*

## Abstract

**Simple Summary:**

Promoters are DNA sequences where the process of transcription starts. They can work constitutively or be controlled by environmental signals of different types. The quantity of proteins and RNA present in yeast genetic circuits highly depends on promoter strength. Hence, they have been deeply studied and modified over, at least, the last forty years, especially since the year 2000 when Synthetic Biology was born. Here, we present how promoter engineering changed over these four decades and discuss its possible future directions due to novel computational methods and technology.

**Abstract:**

Synthetic gene circuits are made of DNA sequences, referred to as transcription units, that communicate by exchanging proteins or RNA molecules. Proteins are, mostly, transcription factors that bind promoter sequences to modulate the expression of other molecules. Promoters are, therefore, key components in genetic circuits. In this review, we focus our attention on the construction of artificial promoters for the yeast *S. cerevisiae*, a popular chassis for gene circuits. We describe the initial techniques and achievements in promoter engineering that predated the start of the Synthetic Biology epoch of about 20 years. We present the main applications of synthetic promoters built via different methods and discuss the latest innovations in the wet-lab engineering of novel promoter sequences.

## 1. Introduction

Synthetic biology is a new branch of biology—whose birth can be set to January 2000 with the publication of the first two *synthetic gene circuits* [1,2]—that aims to standardize and modularize the design and engineering of biological circuits that confer new, useful functions to living cells [3].

Among other applications, genetic circuits are constructed to produce high-value compounds, such as drugs, on an industrial scale [4,5]. To this aim, the yeast *Saccharomyces cerevisiae* appears to be one of the best circuit chassis [6]. The baker’s yeast has a well-annotated genome that can be manipulated in a relatively easy way. Therefore, *S. cerevisiae* cells are turned into *factories* for the production of such valuable chemicals [7,8,9].

Synthetic gene circuits are made of transcription units (TUs) that, in eukaryotes, are composed of three different standard biological parts: promoters, coding sequences (CDSs), and terminators. Inside a circuit, TUs interact via the exchange of proteins or RNA molecules encoded into their CDSs. Initiation and control of transcription occur at the promoter level. Therefore, within a genetic circuit, promoters shall be chosen and engineered carefully to obtain the necessary amount of proteins (and RNAs) that make the circuit work as desired.

Endogenous promoters are characterized by different strengths (i.e., transcription initiation rates) and are divided into two classes: *constitutive* and *inducible*. Constitutive promoters provide relatively stable protein/RNA expression levels under different cell-culture conditions [10]. Inducible promoters initiate gene expression in the presence or absence of certain kinds of molecules (e.g., copper and methionine, respectively) or under particular conditions, such as hypoxia. To modulate gene expression, both constitutive and inducible promoters have been used as templates to engineer new synthetic promoters [11,12,13].

In this review, we describe the different ways synthetic promoters have been built and used in the yeast *S. cerevisiae* over the last forty years. First, we introduce the general structure and working of promoters in budding yeasts. Then, we discuss the engineering of hybrid constitutive promoters. Finally, we illustrate the implementation of regulated promoters and the interactions with their corresponding (native or artificial) transcription factors (TFs). Applications of the diverse synthetic promoters are presented as well.

## 2. The Structure of *S. cerevisiae* Promoters

A promoter is a DNA sequence that enables transcription initiation. The structure of a promoter is specified by the transcription start site (TSS) and the position, relative to the TSS, of various protein-binding sequences. To design promoters for synthetic biology, it is essential to understand how the promoter structure influences promoter strength and performance [14].

*S. cerevisiae* RNA polymerase II-dependent promoters are characterized by three main elements: the upstream activating sequence (UAS), the TATA box, and the transcription start site [15]. Each of these structural motifs can be present in more than a single instance along the same promoter sequence. It should be noted that a large number of promoters in the yeast *S. cerevisiae* do not have a TATA box and are recognized and bound by RNA polymerase II via other structural features (such as a DNA bend) [16]. However, *TATA-less* promoters are out of the scope of this review since they have been used seldom in synthetic gene circuits.

The upstream activating sequences in *S. cerevisiae*, like the enhancers in higher eukaryotes, are bound by activator proteins that recruit to the promoter RNA polymerase II together with the general transcription factor [17]. UASs are located ~100–1400 nucleotides upstream of the *core promoter*, which starts with the first TATA box and finishes at the end of the 5′UTR (untranslated region) [15,18]. UAS variable number and position affect, in general, the extent of gene expression [19].

The TATA box is a hexamer (though longer consensus sequences have been proposed [20,21,22]) consisting mainly of thymine and adenines—with the possible sporadic presence of a single cytosine or guanine [23]—that can be regarded as the RNA polymerase II binding site. Transcription initiation rate (i.e., the promoter strength) depends also on the actual TATA box sequence [24,25].

The location and activity of the transcription start sites are constrained by the position of the TATA boxes [26]. Indeed, a TATA box can activate only TSSs that are placed from 40 up to 120 nt downstream [27]. Moreover, the region around the TSS is characterized by a low content of cytosines and guanine, which corresponds to a reduced nucleosome occupancy [20].

Among the natural promoters in budding yeast, the *CYC1* constitutive promoter (pCYC1—throughout the whole paper we will refer to promoters by using the letter “p” in front of the name of the gene they precede in the yeast genome), which leads the expression of iso-1-Cytochrome C, has been studied deeply and is now well characterized [27,28,29,30]. For this reason, it was used as a template to build new regulated synthetic promoters [31].

pCYC1 contains two UASs, three TATA boxes, and at least six TSSs [27,29,30]. UAS1 is centered, approximately, around position −265, whereas UAS2 overlaps position −229. Deletions along pCYC1 sequence showed that UAS2 alone cannot drive *CYC1* gene transcription in glucose-containing media, whereas in the presence of lactate, UAS1 and UAS2 contributes almost equally to *CYC1* expression [28].

**Figure 1:**
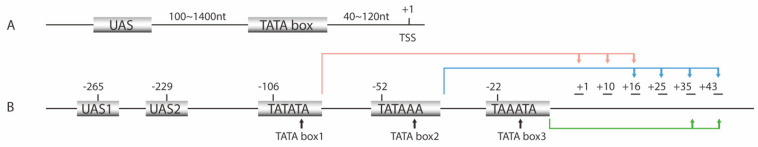
Promoters in *S. cerevisiae*. (**A**) General structure of *S. cerevisiae* promoters. With respect to the TATA box, UASs are located between 100 and 1400 nt upstream, whereas TSSs lie from 40 up to 120 nt downstream. (**B**) The structure of the yeast *CYC1* promoter. Two UASs are present upstream of three TATA boxes, whose sequences are reported in the figure, that activate at least six TSSs [27].

TATA boxes start at position −106, −52, and −22. They activate, respectively, 3, 4, and 2 transcriptions start sites among the six that have been reported so far (see Figure 1).

## 3. Hybrid Promoters

In the yeast *S. cerevisiae*, hybrid promoters are built by merging DNA *elements*—from distinct natural constitutive and inducible promoters—that play diverse roles in transcription. A traditional way to construct hybrid promoters requires combining the upstream activating sequence(s) of one promoter with the core sequence of a different promoter [32]. This technique predated the Synthetic Biology era since, already in 1982, Guarente et al. [31,33] localized the binding region of the GAL4 protein by replacing the *CYC1* promoter UASs with a 365-nt-long sequence from the *GAL10* gene (later referred to as *GAL1,10* intergenic region). The same hybrid promoter was used, a few years later, for studying yeast fermentation [34] and expressing viral proteins in *S. cerevisiae* [35].

**Figure 2:**
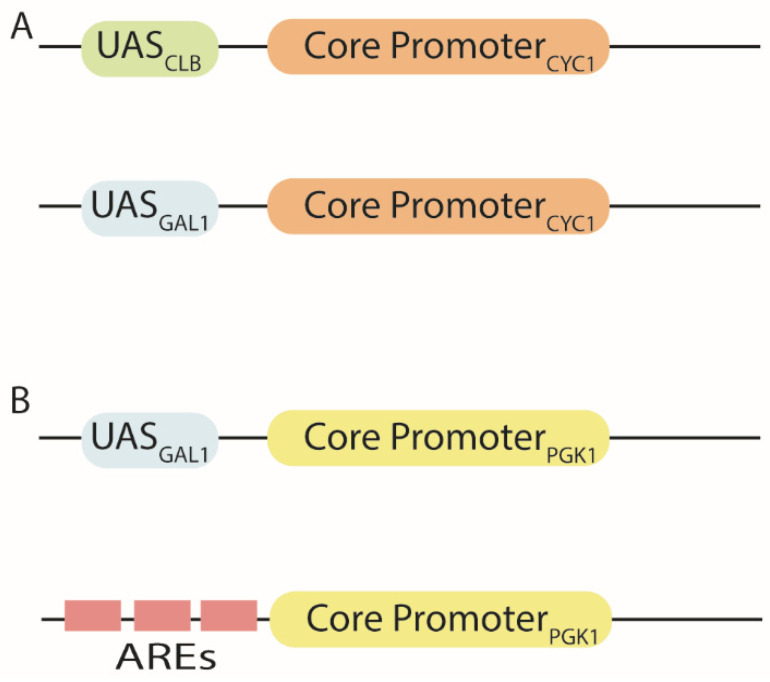
Hybrid promoters. (**A**) The *CYC1* core promoter was merged to different UASs to construct either constitutive or inducible (here galactose-responsive) hybrid promoters [39]. (**B**) The *PGK1* core promoter was turned, as well, into a galactose-inducible hybrid promoter when preceded by the UAS from pGAL1. The substitution of UAS_GAL1_ with androgen-responsive elements (AREs) led to a new hybrid promoter induced by testosterone [41].

Libraries of hybrid promoters are built to both deepen our knowledge of the general promoter activity [14,36,37,38] and achieve different levels of (constitutive/inducible) gene expression. Blazeck et al. [39] generated one of such libraries by fusing between one and three UASs from the constitutive pCLB2 (CycLin B), pCIT1 (CITrate synthase), and pTEF1 (Translation Elongation Factor) to the core minimal pLEU2 (LEUcine biosynthesis), pGPD (Glycerol-3-Phosphate Dehydrogenase), and pCYC1 (Cytochrome C). In this way, they achieved a 2.5-fold increase in the transcriptional activity of pGPD—the strongest constitutive promoter in *S. cerevisiae*. Furthermore, by fusing the upstream activating sequences of the widely employed galactose-inducible *GAL1* promoter to the *CYC1* and *LEUM* core promoters (with the addition of other short DNA motifs) they made a library of galactose-inducible hybrid promoters that spanned an about 50-fold dynamic range in gene expression (see Figure 2A). Interestingly, the fusion of the UAS from pGAL1 to the same *GAL1* promoter enhanced the strength of pGAL1 itself by roughly 15%.

Similarly, hybrid *GPD* promoters were generated by inserting, in variable number and position, the *GAL1,10* intergenic region inside the pGPD sequence. Hybrid *GPD* promoters turned out to be weaker than the native pGPD in glucose-containing media. However, gene expression was 150- to 200-fold induced in the presence of galactose. These hybrid promoters found application in the controlled production of the human immune interferon-gamma (IFN-gamma) whose accumulation provokes toxicity effects into yeast cells [40]. Galactose-inducible hybrid promoters were constructed also by Purvis and co-authors [41] by substituting the UAS of the *PGK1* promoter with the *GAL1,10* intergenic region. It is worth mentioning that, in this work, pPGK1 was turned into a testosterone-responsive promoter as well after replacing its UAS with androgen-responsive elements (see Figure 2B).

Hybrid promoters were engineered to respond to a variety of other signals. For instance, a tryptophan-inducible hybrid promoter was first built in 1999 by placing the tryptophan-responsive UAS of the *ARO9* promoter upstream of the *CYC1* core promoter [42]. More recently, pARO9 was modified by increasing the copies of its own UAS [43]. Furthermore, a whole library of tryptophan-inducible hybrid promoters was generated more recently [44]. Here, a minimal UAS sequence from pARO9 was placed, in variable numbers, upstream of both full length (pCYC1 and pHXT7) and minimal (pLeuMin and CORE1 [22]) promoters.

Other hybrid promoters are induced by particular cellular *stress* conditions such as a temperature increase (heat-shock [45]), oxidative stress [46], variation in pH [47], and lack of glucose [48].

## 4. Promoter Sequence Modification

**Figure 3:**
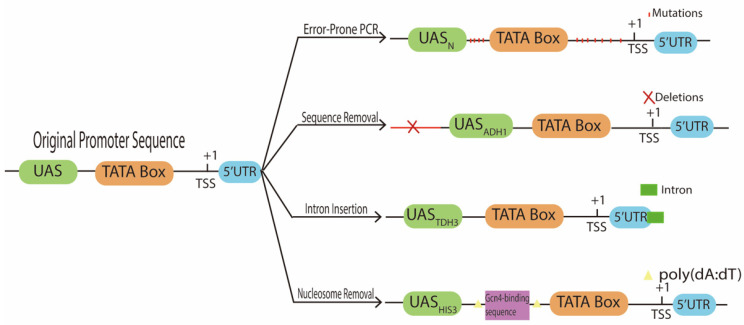
Synthetic promoters obtained by modifying the sequence of native promoters. Common methods to alter promoter sequence and activity are: point mutations via error-prone PCR [11], removal of functionless sequences [49,50], intron insertion along the 5′UTR [51], and nucleosome removal [52].

Hybrid promoters have been, mainly, engineered by combining UASs (or other binding sites for yeast-endogenous activators) and core promoters. Alternatively, synthetic promoters have been constructed by changing the sequence of native yeast promoters via mutations, deletions, nucleosome removal, and intron insertion (see Figure 3).

Back in the nineties of the last century, shortened variants of the constitutive *ADH1* promoter proved to enhance gene expression [49,50]. Later on, a collection of 11 mutants of the constitutive *TEF1* promoter was generated via error-prone PCR [11,53]. The strength of the new synthetic promoters varied from 8% to 120% with respect to the original pTEF1. A similar approach was followed by Du and co-authors [54] to make a library of cellulose-utilizing and xylose-producing pathways. Each pathway contained a mutated version of three *S. cerevisiae* promoters (pTEF1, pPDC1, and pENO2). Error-prone PCR was adopted also to generate a variant of the pheromone responsive *FUS1* promoter, termed pFUS1J2, that showed almost undetectable basal expression and higher gene expression upon induction. pFUS1J2 found application in a positive feedback loop [55] and an RNAi-dependent quorum sensing system [56].

Nucleosome removal is a strategy to increase promoter activity. According to it, Raveh-Sadka et al. [52] built a library of 70 synthetic promoters by modifying the yeast native *HIS3* promoter with the insertion of the Gcn4-binding sequence flanked by two poly(dA:dT) sequences (one 10-, the other 17-nucleotide-long) that highly disfavored nucleosome formation. Moreover, Curran and co-authors [57] improved the strength of some constitutive yeast promoters (pCYC1, pHIS5, pHXT7, and pTEF1) by eliminating nucleosomes from their sequences. Nucleosome occupancy along a promoter sequence was calculated via the NuPoP algorithm [58]. Then, mutations were carried out to remove nucleosomes without introducing TATA boxes or known TF operators. By iterating the procedure, the number of nucleosomes was lowered at each step with a consequent increase in promoter strength. Furthermore, new synthetic promoters were designed by starting from two templates containing minimal motifs (e.g., a consensus TATA box, the 5′UTR of the *GPD* promoter, and binding sites for glycolytic TFs) joint by random DNA sequences. By applying the NuPoP algorithm to these synthetic sequences, promoters with higher strengths (up to 20-fold than the corresponding template) were computed. These new synthetic promoters had minimal homology with those in the *S. cerevisiae* genome [57].

Another, different way to affect promoter activity demands the insertion of intron sequences along the 5′UTR, as illustrated in [51]. This technique, however, is context-dependent and can have repercussions on mRNA translation. Myburgh et al. [59] reported an increase in amylase and ethanol production by placing the RPS25A intron at the end of the 5′UTR of native yeast promoters.

A different strategy to design synthetic inducible promoters was illustrated in [60] and required the detection and optimization of natural inducible-promoter. In this work, RNA-seq was carried out to find out which genes were overexpressed from cells grown in a solution containing 1-butanol. Four genes were identified and their putative promoters (i.e., the sequence up to 1000 nt upstream of the START codon) were considered for further analysis. Only two of them (pNRP1 and pSHQ1) proved to react specifically to 1-butanol. They were, finally, shortened to the minimal length able to respond to 1-butanol.

**Figure 4:**
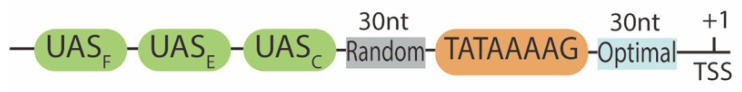
Minimal synthetic promoter design. The 8-nt-long TATA box is separated by three 10-nt-long UASs through a random spacer (30 nt). The TSS is placed 30 nt downstream of the TATA box (the sequence *Optimal* was designed to prevent nucleosome formation). This synthetic promoter, which is almost as strong as pGPD, has an overall length of only 116 nt [22].

Instead of just modifying promoter sequences, Redden and co-authors [22] presented a procedure for the modular construction of short, minimal, synthetic promoters. Upon screening two large promoter libraries (15 and 1.3 million sequences), a small number of highly functional, context-independent, core promoter elements (9) and UASs (6) were found. Minimal core promoters were built by joining an 8-nt-long TATA box (TATAAAAG) and a TSS (with consensus sequence: A(Arich)_5_NYAWNN(Arich)_6_). They were separated by 30 nt only. UASs, whose length was 10 nt, were placed upstream of the TATA box. Between them, a 30-nt-long spacer randomly generated, was inserted. By using three UASs, a core promoter, and the 30-nt-long spacer in between, a synthetic promoter almost as strong as pGPD but only 116-nt long (versus the 655 nt of pGPD) was built (see Figure 4). These short synthetic promoters can reduce considerably the burden associated with genetic circuits.

Different short synthetic promoters were obtained in [61]. This work presents a library of artificial promoters, of length variable between 69 and 129 nucleotides, spanning a 20-fold activity range.

Novel synthetic constitutive core promoters, of moderate strength, were also generated by placing the −16…+71 region of pCYC1 downstream of either natural or synthetic *S. cerevisiae* terminators [62]. The sequence from pCYC1 contains only transcription start sites—within the whole 5′ untranslated region. Thus, it was termed pCYC1noTATA. Terminators, in *S. cerevisiae*, are characterized by three short sequences: the efficiency element, the positioning element, and the poly(A) site [63]. The efficiency element is at least six-nucleotide long and mainly contains thymines and adenines [64]. Therefore, it mimics a TATA box if placed from 40 up to 120 nucleotides upstream of a TSS. The strongest promoter engineered in this work contained the potent *DEG1* terminator [65], the weakest made use of the *CYC1* terminator.

## 5. Synthetic Promoters Regulated by Bacterial Proteins

**Figure 5:**
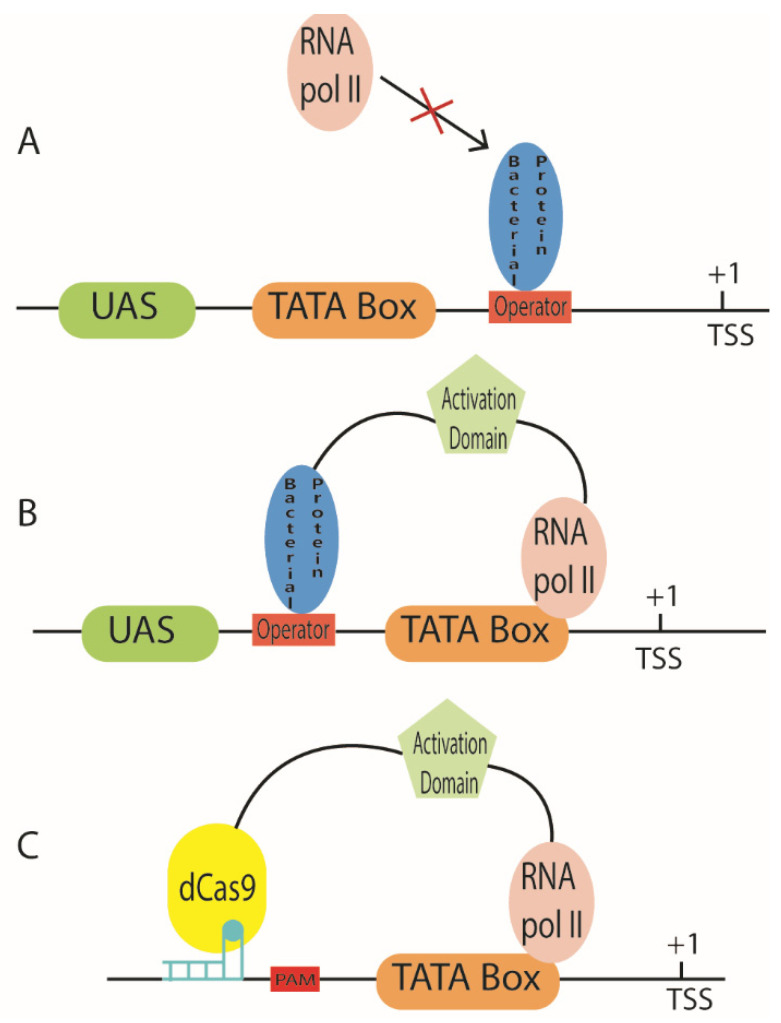
Promoters regulated by bacterial proteins. (**A**) Repressed promoters demand to place the operator of a bacterial protein between the TATA box and the TSS. (**B**) To build activated promoters, operators shall be placed upstream of the TATA box. Moreover, a bacterial protein behaves as a yeast activator upon fusion to an activation domain. (**C**) The CRISPR-dCas9 system can be used to either activate (as in the figure) or repress transcription. The sgRNA binds a complementary sequence, along the promoter, that is followed by a protospacer adjacent motif (PAM).

Transcription factors (repressors and activators) are proteins that wire together two or more TUs inside a genetic circuit. They regulate the transcription initiation rate of their target promoters upon binding specific operators. Synthetic gene circuits should be *orthogonal* to their chassis i.e., they should not interfere with the molecular biology of the host cell. Therefore, to have orthogonality, the transcription factors expressed by a circuit should not bind DNA outside of the synthetic gene network. This is achieved, in *S. cerevisiae*, by using bacterial proteins as TFs and inserting their operators within the sequences of the circuit promoters that need to be repressed or activated (see Figure 5A,B).

In the following, we describe yeast synthetic promoters regulated by eight different kinds of bacterial proteins. Importantly, each protein can be controlled by an environmental signal such that gene expression is induced or inhibited by either adding/removing chemicals to/from the cell solution or growing cells under particular conditions.

### 5.1. LexA-Regulated Promoters

LexA is an *E. coli* repressor protein that binds a rather long operator (lexO, 41-nucleotide long) organized in two subunits separated by a single base pair [66,67]. The first yeast artificial promoter regulated by LexA was built in the early eighties of the last century. Brent and Ptashne [68] turned pGAL1 into a repressed promoter by inserting one or two shorter LexA binding sites (22-nt long) between the UAS and TATA box. In a successive work [69], they placed the same 22-nt-long LexA binding site at different locations upstream of the TSS of pGAL1 and pCYC1. Moreover, LexA was fused to the activation domain of the GAL4 yeast protein (LexA-GAL4*AD*) and worked as an activator for the two engineered promoters. More yeast synthetic activators were, later on, constructed by fusing either the GAL4 DNA binding domain (GAL4*DBD*) or the whole LexA to activating sequences from *E. coli* such as B42 [70] and the highly active B112 [71]. In particular, the chimeric protein LexA-B42 was the first example of a completely orthogonal yeast activator, since it was made of two bacterial protein domains [72].

In 1992, LexA served as a DBD for a chimeric repressor that contained also the chromatin remodeling factor Ssn6 [73]. One year later, Louvion et al. [74] showed that the hormone-binding domain of the human estrogen receptor—HBD(hER)—worked properly in *S. cerevisiae* and could be adopted to control transcription via molecules of beta-estradiol. They made a chimeric activator by fusing together the GAL4DBD, the HBD(hER), and the VP16 activation domain from the Herpes simplex virus [75]. In the absence of beta-estradiol, HBD(hER) is sequestered into the cytoplasm by the heat-shock protein Hsp90. In contrast, when beta-estradiol is added to cell culture, the progesterone strongly binds HBD(hER) and prevents any further interactions with Hsp90, such that the whole chimeric protein—also referred to as GEV—translocate into the nucleus [76]. A planar aromatic hydrocarbon biosensor was constructed, in 1993, by Carver and co-authors [77] in what can be regarded as a Synthetic Biology work *ante litteram*. A synthetic promoter was engineered by replacing the UAS of pGAL1 with eight copies of the lex operator. In this way, the promoter was activated by a chimeric protein made of LexA fused to the aryl hydrocarbon receptor (AHR), which contains an activation domain. Like HBD(hER), AHR interacts with Hsp90 in the absence of its ligand(s). Only in the presence of alpha- or beta-naphthoflavone, LexA-AHL was able to translocate into the nucleus and stimulate the production of the LacZ reporter protein.

A similar structure for a synthetic promoter was adopted in [78] for the construction of a memory circuit in *S. cerevisiae*. In this work, the minimal *CYC1* promoter was preceded by eight lex operators bound by LexA-VP64 (i.e., an activation domain made of four VP16 subunits).

Synthetic promoters based on a minimal pCYC1 (where only the TATA box at position −52 was kept) extended with a variable number of lexO (1 to 8) were used in [79] as targets for chimeric activators consisting of LexA, HBD(hER), and an activation domain among B42, B112, GAL4AD, and VP16. Moderate expression of LexA-HBD(hER)-B112 induced a considerable fluorescence signal from a minimal pCYC1—preceded by (at least) four lexO sites—in the presence of up to 2 μM beta-estradiol. In a similar way, Dossani and co-authors [80] used the chimeric activator LexA-HBD(hER)-VP16 to enhance fluorescence production from a library of 154 synthetic promoters. These promoters were built by adding 1–3 LexA binding sites (chosen among four possible ones [69]) in front of the core promoter (here defined as the sequence of either 100 or 250 nucleotides upstream of the START codon) of 10 *S. cerevisiae* genes. Fluorescence, from each promoter, was measured at low concentrations of beta-estradiol (up to 100 nM). The core pGAL1 showed both the highest response (maximal fluorescence at 10 nM beta-estradiol) and—together with the core pSPO13—the best inducibility (on/off ratio).

Rantasalo and co-authors [81] described a LexA-dependent fixed-gain bidirectional amplifier structure. Each amplifier was made of two core promoters (200-nt long) divided by a variable number (from 1 to 8) of short LexA binding sites (16-nt long). Both promoters were activated by LexA fused to either VP16 or B42. The best performing amplifier consisted of the core *ENO1* promoter on the antisense strand and the core *PGK1* promoter on the sense strand.

In a following work by the same group, more bacterial proteins were tested both as activators and repressors of engineered core promoters from *S. cerevisiae* and filamentous fungi (*Trichoderma resei* and *Aspergillus niger*) [82]. SrpR, PhlF, TetR, and Bm3Rl turned out to be effective both as activators (upon fusion to VP16) and repressors (bare protein). In contrast, LexA and TarA worked only as activators, whereas LacI functioned only as a repressor (interestingly, LacI-VP16 was previously reported to be a potent activator in mammalian cells [83]). The synthetic activator Bm3R1-VP16 was further employed—within an architecture derived from [81]—to build a *universal* gene-expression system for fungi that was validated in six yeast species and the two filamentous fungi mentioned above [84].

### 5.2. TetR-Regulated Promoters

The bacterial repressor protein TetR, in its wild-type configuration, binds the DNA at a 19-nt-long sequence, the tet operator—tetO. Antibiotics belonging to the family of tetracycline dock to TetR and induce a change in its structure that prevents any further DNA binding [85]. Gossen and Bujard [86] engineered a chimeric activator, which proved to work in eukaryotic cells, by fusing TetR to the VP16 activation domain. Since tetracycline precludes gene expression from TetR-VP16, the overall system tetracycline—TetR-VP16 is usually referred to as *tet-off*. In a later work, Gossen and co-authors [87] constructed a *reverse* version of TetR (indicated as TetR’) that binds the DNA only in the presence of tetracycline. As a consequence, the tetracycline—TetR’-VP16 system was termed *tet-on*.

Garí et al. [88] realized two synthetic promoters by placing either two or seven tetO sites upstream of a minimal *CYC1* promoter. Gene expression was enhanced by fusing one or two copies of VP16 to TetR. Moreover, the insertion of a linker between TetR and VP16 (and also between two VP16 domains) was a simple way to further increase the potency of tet-off-based systems. In a later work from the same lab, Belli’ and co-authors [89] gave a more detailed characterization of tetO-containing promoters to achieve either activation or repression of gene expression. In particular, TetR and TetR’ were fused to two different *S. cerevisiae* repression moieties, Ssn6 and Tup1, the former providing a tighter transcriptional control. In a more recent work [90], the promoters from [88] were reduced, in length, to contain a single tetO. Moreover, variants of the tet operator, engineered via point mutations, led to an over 100-fold range of luciferase expression under the action of TetR-VP16. These new TetR-binding sites were applied in the re-construction of the pathway for the antioxidant lycopene production in *S. cerevisiae*. Remarkably, Mnaimneh et al. [91] analyzed the effect of the changes in the expression profile of almost 700 essential genes in *S. cerevisiae* via promoters down-regulated via a cassette of seven tet operators.

The repressive action of the bare TetR on gene expression in *S. cerevisiae* was characterized in [92]. Seven synthetic repressed promoters were built by inserting from one up to three tetO sites at three different positions between the TATA box and the TSS of the *GAL1* promoter. The repression level due to TetR increased with the operator number. Moreover, higher repression was achieved by placing the operators closer to the TATA box rather than to the TSS. One of the promoters containing two tetO sites was the basis for the construction of a library of 20 more repressed promoters that covered a wide range of gene expression and inhibition levels. This was achieved by mutating randomly the region around the two tet operators [93]. These new promoters were used inside an incoherent type I1 FFL (feed-forward loop) and a genetic timer.

A rare example of *TATA-less* promoter engineering was given in [94] where two tetO sites were inserted along the core sequence of the *S. cerevisiae PFY1* promoter. pPFY1 displays, instead of the TATA-box, a poly-T sequence that provokes a DNA bend probably responsible for the access of RNA polymerase II to the core promoter. The new tetracycline-controlled promoter (pPFY1i) showed very low basal fluorescence expression in the absence of tetracycline and reached around 75% of pPFY1 fluorescent level upon induction with tetracycline. Both pPFY1 and pPFY1i were used as targets for TALORs (transcription activator-like orthogonal repressors; see Section 6.2 below for information about TALEs). A protein, named P-TALOR, was designed to target a 17-nt-long sequence along the original pPFY1. A second TALOR, termed I-TALOR, bound a 16-nt-long “operator” on pPFY1i. The two binding sites shared only 9 nucleotides i.e., they were chosen to avoid crosstalk between the TALORs. As expected, the two TALORs were orthogonal to each other and reduced, drastically, fluorescence expression, with I-TALOR showing a slightly higher affinity than P-TALOR towards its own target site.

### 5.3. LacI-Regulated Promoters

In *E. coli* cells, the Lac repressor (LacI) binds three operators with different affinities. An ideal, 20-nucleotide-long operator (lacO) sequence has been proposed by Oehler and co-authors [95]. LacI action on the DNA is regulated by IPTG (isopropyl b-D-1-thiogalactopyranoside). More precisely, IPTG induces conformational changes on LacI that prevent its binding to the DNA.

The first LacI-repressed synthetic promoter in *S. cerevisiae* was engineered by Grilly and co-authors [96] that modified the strong *ADH1* promoter with the insertion of a single lacO downstream of the TATA box. Two copies of this synthetic promoter, termed pADH1-i, were employed to drive, in the presence of IPTG, the synthesis of the bacterial proteins ClpX and ClpP that dimerize to form the ClpXP protease. In this circuit, the green fluorescent protein gene, responsible for the production of an output signal, was fused to the ssrA tag that is recognized by ClpXP. Therefore, in the absence of IPTG, the network expressed green fluorescence, whereas, in the presence of IPTG, ClpXP degraded the tagged GFP with a consequent decrease in fluorescence.

Ellis and co-authors [93] constructed—together with the library of TetR-regulated promoters previously mentioned—a library of 20 *GAL1*-based LacI-repressed promoters. They employed these promoters as components of genetic timers. Moreover, they built a synthetic promoter hosting tetOs in tandem together with a single lacO. The promoter, highly derepressed only in the presence of both tetracycline and IPTG, was a part of an I1 FFL.

Similar promoter configurations, based on simultaneous repression by TetR and LacI, were engineered in [97] to mimic logic functions. In this work, synthetic hybrid bipartite promoters were realized by combining a segment from the *CYC1* promoter (containing the TSSs) with a portion of the *VPH1* promoter (enclosing the TATA box). The two promoter pieces were joined by a short spacer where one or two binding sites for bacterial proteins (TetR, LacI, and LexA—the latter fused to HBD(hER)) were placed to achieve repression of transcription. Single-input YES and NOT gates together with two-input AND, N-IMPLY, and OR gates were generated by changing the number and the relative position of the operators, beside the copy (integration) number of the plasmid containing the bipartite promoter.

A dual-mode promoter, i.e., a promoter that can be both activated and repressed, was engineered by Mazumder and McMillen [98]. A weak minimal *CYC1* promoter was modified with the insertion of a lac operator downstream and five binding sites of the human androgen receptor upstream of the TATA box at position −52. The human androgen receptor binds the DNA only in the presence of testosterone and works as an activator. In the absence of IPTG, LacI binds the corresponding operator and prevents fluorescence expression. If cells are induced only with IPTG, the fluorescence level stays low. Thus, only in the presence of both chemicals, the promoter expresses the green fluorescence protein in high quantity—mimicking, in this way, an AND gate. A different dual-mode promoter has been implemented [99] and computationally optimized [100] by using LacI as a repressor and TetR-VP16 as an activator.

### 5.4. XylR-Regulated Promoters

Xylose is a sugar abundant in lignocellulosic lysates and used, by microbes, in the production of several chemicals [101]. Xylose-responsive transcription factors (XylRs) are present in many bacterial species and bind the DNA only in the absence of xylose. Together with their operators, XylRs have been used in the construction of xylose-inducible biosensors in yeast.

Teo and Chang [102] cloned, in *S. cerevisiae*, XylR from three different bacteria. Each XylR targeted a synthetic promoter made of the UAS from pTEF1 and the core *GAL1* promoter was modified with a xyl operator (xylO) downstream of the TATA box. Since the sequence of xylO is not conserved among bacterial species, a different hybrid promoter was constructed for each of the three XylRs. By following an analogous strategy, Wang and co-authors [103] built xylose biosensors by means of three more XylR proteins. They were tested on four synthetic promoters, each based on a reduced *GPM1* promoter that kept its own UAS. Three promoters contained a single xylO located 19 nucleotides downstream or 1 nucleotide upstream of the TATA box, or 1 nucleotide downstream of the UAS. In the fourth promoter, two xylOs were employed: one downstream of the UAS, the other upstream of the TATA box. Furthermore, after deriving a consensus sequence for xylO, a library of synthetic promoters was realized through PCR with primers containing degenerate operator sequences. The new operators were located upstream of the TATA box.

Hector and Mertens [104] constructed another xylose-responsive synthetic promoter by starting from the constitutive *TEF* promoter of the filamentous fungus *Ashbya gossypii*. The original promoter sequence was modified with the removal of one of the two TATA boxes and the addition of one or two xyl operators in the proximity of the only TATA box left. XylR was fused to the yeast chromatin remodeling protein Ssn6 to enhance the repression of transcription. The best performance in terms of induction of *lacZ* expression—up to 25-fold—was obtained when the TATA box was flanked, at each side, by a xyl operator.

A different xylose biosensor was engineered in [105] by exploiting a *reverse* XylR from *E. coli* (EcXylR^R^) that binds the DNA only in the presence of xylose. EcXylR^R^ was fused to the strong composite VPRH (VP64, p65, Rta, and HSF1) activation domain. The original biosensor was implemented in the yeast *Yarrowia lipolytica*. Here, four synthetic promoters were built by placing a xylO upstream of the TATA box of the complete, strong *TEF* promoter, and of three core promoters obtained from the constitutive *TEF, hp4d*, and *ACC1* promoters. The synthetic promoter based on the *TEF* core promoter was the most performant in terms of on-to-off ratio with a 20.9-fold gain upon xylose induction. This biosensor configuration was then ported to *S. cerevisiae* cells. With respect to the background fluorescence, the circuit returned a 43.3-fold higher signal in the presence of xylose.

### 5.5. FadR- and FapR-Regulated Promoters

Fatty acid (and fatty acyl-CoA) sensing devices based on the FadR repressor from two different bacterial sources (*E. coli* and *Vibrio cholerae*) were realized in [106]. FadR is active, i.e., able to bind its operator, in the absence of fatty acids. In this work, a fluorescent signal was driven by synthetic promoters realized by inserting either one or three FadR operators between the TATA box and the TSS of the *GAL1* promoter. Strong fluorescence repression was achieved with the *E. coli* FadR binding a 3-operator-containing pGAL1. Such a biosensor can be adopted to regulate protein expression—and, thus, balance the cellular metabolism—according to the availability of fatty acid intermediates inside yeast cells. In a later work by the same lab, Teo and Chang [107] modified the pGAL1-based synthetic promoter by inserting three *tandem* operators of the *E. coli* FadR along the core promoter sequence and replacing the original UAS with that of either constitutive (*TEF1*, *PGK1*, and *HXT7*) or inducible (*CUP1* and *PHO5*) yeast promoters. The first three new synthetic promoters were used to detect fatty acids in media containing glucose, whereas the last two promoters permitted to realize Boolean gates responding to fatty acids and either the presence of copper (AND gate) or the absence of phosphates (N-IMPLY gate).

Malonyl-CoA is a basic building block for the biosynthesis of value-added compounds such as fatty acids, polyketides, and flavonoids. Li and co-authors [108] engineered a malonyl-CoA biosensor, in *S. cerevisiae*, by means of the protein FapR (from *B. subtilis*) that binds a 34-nt-long operator (fapO) only in the absence of malonyl-CoA. A synthetic promoter repressed by FapR was built on the yeast *GPM1* promoter. Only one UAS was kept along the promoter sequence and either 1 or 2 fapO sites were placed upstream of the TATA box. The best biosensor configuration (lowest leakage and highest dynamic range) was achieved with *fapR* gene on an episomal plasmid (high protein number) and a single fapO in the engineered pGPM1 (4.17-fold gain). David and co-authors [109] constructed another malonyl-CoA biosensor to regulate a metabolic pathway for the production of 3-hydroxypropionic acid (3-HP). Synthetic promoters were engineered by placing 1 to 3 copies of fapO along pTEF1, in the region between −50 and +50 nt with respect to the TSS. The variant with 3 fapOs led to a 10-fold increase in 3-HP production.

A similar pTEF1-based synthetic promoter was employed in [110] to implement another fatty acid biosensor that made use of the *E. coli* FadR repressor. FadR operators replaced fapO sites on the synthetic pTEF1 promoters in [109]. This biosensor was used to screen a gene library for the enhancement of acyl-CoA concentration in yeast. Upon overexpression, three genes (*RTC3*, *GGA2*, and *LPP1*) were shown to lead to an about 80% increase in the level of fatty alcohols.

The work in [109] was continued by Dabirian and co-authors [14,111]. Here, both constitutive (pTDH3, pCCW12, and pTPI1) and glucose-inducible promoters (pADH2 and pMDH2) were turned into FapR-repressed (i.e., malonyl-CoA induced) promoters with the insertions of a different number of fapO sites (up to three) at four different positions (just upstream and downstream of the TATA box and the TSS). The sites close to the TATA box allowed, as expected, higher repression. A remarkable dynamic range in gene expression (95-fold) was achieved by modifying pCCW12 with a single fapO downstream of the TATA box. Since the insertion of operators along the core promoter sequence caused a decrease in the promoter activity (as also pointed out, recently, in [112]), the authors hypothesized that a promoter could be repressed, without losing its native strength, by enhancing the action of the repressor and moving the operators upstream of the core promoter. The *TEF1* promoter re-engineered with three fapO sites upstream of the TATA box conserved the strength of the native *TEF1* promoter and was 5.4-fold repressed by FapR fused to the Mig1 repression domain.

A novel design for malonyl-CoA, fatty acid, and xylose biosensors was presented in [113]. These biosensors, differently from those described above, were based on synthetic activated promoters. The malonyl-CoA biosensor was the first to be realized. Its best configuration consisted of the chimeric activator FapR-Med2 (an AD from the mediator complex) acting on the weak *LEU2* promoter modified with a fapO site 57 nt upstream of the TATA box. This biosensor gave a 53.5-fold increase in fluorescence expression in the absence of the malonyl-CoA and an 82% decrease in the presence of 20 uM cerulenin (which provokes an increase in malonyl-CoA concentration). Upon replacement of fapO with either a FadR or a XylR binding site in the synthetic promoter, the circuit was turned into a biosensor for either fatty acid or xylose.

### 5.6. MetJ- and BenM-Regulated Promoters

SAM (S-adenosylmethionine) is a methyl donor for the modification of multiple molecules such as DNA, RNA, and proteins. Upon binding SAM, the *E. coli* repressor protein MetJ is enabled to adhere to the DNA at its operator sequence, metO.

A SAM biosensor was engineered, in *S. cerevisiae* by, employing a novel synthetic activator, MetJ-B42, targeting a synthetic *CYC1* promoter hosting metO upstream of the TATA box (metO-pCYC1) [114]. metO-pCYC1 was later modified with a tetO close to the TSS. In this way, the biosensor was turned into an AND gate, responding to both SAM and doxycycline, that was used to screen a plasmid library looking for genes that could enhance SAM production.

Skjoedt and co-authors [115] built biosensors for chemicals binding LysR-type transcriptional regulators (LTTRs). The best performing transcription factor used in this work was BenM, from *Acinetobacter* sp *ADP1*, which responds to CCM (cis,cis-muconic acid). In bacteria, BenM is an activator that binds the DNA independently of its ligand. In the presence of CCM, BenM induces a structural change in the DNA that enhances the binding of RNA polymerase to the promoter. Initially, the author constructed three synthetic promoters based on the yeast *CYC1* promoter. They placed a single BenM operator (benO) upstream of the TATA box at position −106 (T1 variant) or upstream of the TATA box at position −52 (T2 variant). A third configuration (T1-T2) contained both benOs. When BenM was produced under pTEF1, fluorescence increased in T1 (20 folds) and T1–T2 (5 folds) promoter configuration proving that BenM works, as an activator, also in yeast without the need for any AD. By growing the cells in a 1.4 mM CCM solution, however, only a modest enhancement in fluorescence expression was detected. Therefore, the whole system had to be optimized to return a clear response to CCM. To this aim, the best promoter design turned out to be the “209bp_CYC1_BenO_T1”. This synthetic promoter was used to build three more biosensors by replacing benO with the operator of other LTTRs, namely: FdeR (sensing naringenin); ArgP (L-arginine), and MdcR (malonic acid).

## 6. Templates for Synthetic Transcription Factors

The use of bacterial proteins as orthogonal TFs in *S. cerevisiae* demands to modify yeast promoters with the insertion of operators that, usually, reduce promoter activity when placed within the core sequence. To avoid this issue, zinc finger proteins (ZFPs), TAL effectors (TALEs), and CRISPR-Cas have been adopted to engineer new, orthogonal TFs. These three systems allow targeting almost every sequence within a native promoter by customizing a DNA-binding domain (ZFPs and TALEs) or expressing a short RNA molecule (CRISPR-Cas).

### 6.1. Zinc Finger Protein (ZFP)-Regulated Promoters

A zinc finger domain (ZFD) is a structural motif, made of about 30 amino acids, that recognizes and binds a DNA triplet. Adjacent ZFDs constitute the DBD of zinc finger proteins. Synthetic TFs have been constructed by fusing activation or repression domains to either a naturally occurring DBD from zinc finger proteins or a novel DBD obtained by joining from three to six ZFDs. ZF-based DBDs proved to bind their target DNA sequence with high specificity [116].

A minimal *CYC1* promoter sequence was turned into a synthetic activated promoter by placing, upstream of the TATA box, a variable number of either the binding site of the natural murine zinc-finger protein Zif268 [78] or short operators (9–18 nt) for ZFP-based synthetic DBDs [117].

McIsaac and co-authors [118] modified the GEV system [74] by replacing the GAL4DBD with two different Cis2-His2 zinc-finger DBDs: one from Zif268 (the corresponding new synthetic activator was termed Z3), the other designed *de novo* by merging 4 ZFDs (Z4). Z3 and Z4 were built to overcome the problem of the high number of off-target binding sites (over 500) associated with GEV. Z3 had 11 predicted binding sites along the *S. cerevisiae* genome, whereas Z4 was designed to bind only its own operator. Both Z3 and Z4 activated a synthetic promoter made by replacing the 3 UASs of the GAL1 promoter with 6 of their binding sites. Z3 was further characterized in a later work by the same group [119] where it activated different synthetic promoters, based not only on pGAL1 but also on pCYC1 and pDAN1. Interestingly, a vast library of over 327,000 Z3- and Z4-regulated promoter sequences (together with an even larger library of pGPD-based constitutive promoters) was used in a recent work [120] to build a predictive model of promoter activity based on convolutional neural networks (CNN).

### 6.2. TAL Effector-Regulated Promoters

Transcription activator-like effectors (TALEs) are, like ZFPs, a family of proteins characterized by a modular DNA-binding domain [121]. TALEs, as they are found in bacteria, present a DBD organized in a variable number of repeats (from 13 up to 28). Most repeats are made of 34 amino acids (the last repeat is only 20-amino-acid long). The amino acid at position 13 determines the nucleotide recognized and bound by the repeat. Hence, it is called *base-specifying residue* [122]. The DBD of a synthetic TALE-based transcription factor is obtained by joining as many repeats as there are nucleotides in the targeted sequence. As a constraint, only DNA sequences preceded by a thymine or a cytosine are bound by TALE-like proteins.

Besides the TALOR-repressed promoters [94] previously described, another example of synthetic promoters regulated by TAL effectors are given in [123]. Here, 15 TALEs were assembled (10 fused to the GAL4 AD, 5 to VP64). Moreover, several synthetic promoters were generated by placing, upstream of a minimal *CYC1* promoter, a variable number of TALEs’ operators (up to 16) both in forward and reverse sense (their length was 19 nt for the 10 TALEs carrying GAL4D, 20 nt for the other 5). The promoters targeted by TALE-VP64 were bound also by dCas9-VP64-based activators (see below). TALEs outperformed dCas9-VP64. The best TALE-activated promoter showed a 400-fold enhancement, in fluorescence, upon binding of the TAL effectors. The activation efficiency scaled with the operator number and, interestingly, was higher with reverse operators. Fine-tuning was possible by inserting one or two mismatches in the operators, whereas three mismatches, in general, prevented TALEs from binding the DNA.

### 6.3. CRISPR-Cas-Regulated Promoters

CRISPR (clustered regularly interspaced short palindromic repeats)–Cas (CRISPR-associated protein) [124] represents a component of the immune system in bacteria and archaea [125]. Six types (divided into two classes) of CRISPR-Cas have been discovered, so far. Among them, type II is the most largely used in biotechnology since it demands the expression of a single CRISPR-associated protein, usually SpCas9 (i.e., from the bacterium *Streptococcus pyogenes*).

Upon intrusion of foreign DNA, a CRISPR array, made of *spacers* alternated with *direct repeats*, is transcribed into a long pre-CRISPR RNA sequence (pre-crRNA). The spacers are short pieces of DNA that were inserted into the bacterial chromosome after previous infections. The pre-crRNA is processed by the joint action of a further short RNA molecule (named tracrRNA—transactivating crRNA), RNAse III, and SpCas9. As a result, the complex SpCas9-crRNA:tracrRNA is released. The spacers (included in the crRNA) that are complementary to the intruder DNA bind it by base pairing in the proximity of the protospacer adjacent motifs (PAM, which corresponds to the triplet NGG or NAG) and allow SpCas9 to induce a double-strand break in the foreign DNA, which provokes its fast degradation [126]. Jinek et al. [127] showed that the crRNA:tracrRNA structure can be encompassed into a single-guide RNA molecule (sgRNA) that is expressed, in eukaryotes, by an RNA polymerase III-dependent transcription cassette. This finding facilitated the application of CRISPR-SpCas9 to gene editing. Furthermore, the nuclease-deficient SpCas9 protein (dSpCas9), obtained via two mutations (D10A and H841A) along the RuvC and HNH nuclease domains, was adopted for the engineering of new transcription factors [128] of usage in transcriptional networks. Differently from TAL effectors and Zinc Finger proteins, dSpCas9-based transcription factors do not require the assembly of multiple, long DNA sequences to build new DNA-binding domains. In contrast, sgRNAs are simply designed to match a 20-nt-long sequence, preceding a PAM, either within or upstream of a core promoter. Furthermore, activation or repression domains can be fused to dSpCas9 or the sgRNA itself [129] (see Figure 5C). Even though this kind of transcription factor appears to be extremely useful to target and regulate native promoters, it has also been exploited to regulate synthetic promoters constructed *ad hoc*.

Farzadfard and co-authors [130] studied the effect of dSpCas9-VP64 on the minimal *CYC1* promoter. Interestingly, they showed that dSpCas9-VP64 behaves as a repressor if it binds in the proximity and downstream of the TATA box. Moreover, they built synthetic activated promoters by placing a variable number (up to 12) of a chosen 20-nucleotide-long target sequence (termed *a1*) upstream of the TATA box. As expected, fluorescence expression increased with *a1* number, though without a remarkable difference between 3 and 12 *a1* sequences. The configuration with six *a1* sites was utilized into an AND gate where dSpCas9-VP64 expression was conditioned to the presence of galactose in the cell culture and the sgRNA transcription was triggered by tetracycline. Similarly, Machens and co-authors [123] measured the fluorescence induced by dSpCas9-VP64 on a minimal *CYC1* promoter preceded by a variable number (from 2 up to 16) of arbitrarily chosen binding sites (BSs). Fluorescence expression, however, did not scale with the operator number and was, usually, very low for promoter configurations with 16 BSs. The short distance between adjacent BSs (6 nucleotides instead of the 20 in [130]) was indicated as a possible cause of the reduced fluorescence signal.

The tet operator was the target for dSpCas9 in [131]. Seven tet operators were positioned upstream of a minimal promoter driving fluorescence production. TetR’-VP16 induced a high fluorescent signal from this synthetic promoter. However, the addition of dSpCas9 together with a sgRNA complementary to tetO provoked a 115-fold decrease in the cell fluorescence level. Hence, the dSpCas9:sgRNA system proved to have a considerably high affinity towards its DNA target. Other promoters hosting tetOs were employed in [132] to test the efficiency of scaffold RNAs (scRNAs) in *S. cerevisiae*. scRNAs are sgRNA extended, for instance, with one or two MS2 hairpins where the MCP protein, fused to a repression or an activation domain, can bind.

Finally, Gander and co-authors [133] engineered a universal NOR gate starting from a minimal *CYC1* promoter preceded by a UAS from pGPD and modified in the proximity of the TATA box with two target sites complementary to as many sgRNAs. Inputs for the NOR gate were two distinct sgRNAs that formed a complex with dSpCas9-Mxi1 (Mxi1 is a mammalian repressor domain [131]). The output was another sgRNA synthesized through an RGR (hammerhead ribozyme-sgRNA-hepatitis delta virus ribozyme) cassette [134]. NOR gates regulated by diverse sgRNAs could be combined into digital circuits associated with complex Boolean functions.

## 7. Conclusions

The engineering of hybrid *S. cerevisiae* promoters and the usage of bacterial proteins as orthogonal transcription factors in yeast gave important results in the eighties and nineties of the last century. Techniques and methods developed in those years have been inherited by the Synthetic Biology community and are still currently used in the construction of many synthetic gene circuits. Over the years, more bacterial proteins were found to be working in yeast and were used to build, principally, biosensors whenever they interacted with chemicals of various origins or reacted to stress conditions. The real novelty of the last decade was in TF, rather than promoter, engineering with the discovery of TAL effectors and, especially, the CRISPR-Cas systems.

Synthetic Biology, however, aims at the rational, model-driven design of both circuits and DNA components—such as promoters. Mathematical instruments, from System Biology [135,136], have allowed achieving, at least in some cases, quantitative circuit description, though with usually low predictive valence. Big data and machine learning [120] represent a new powerful approach to understand promoter working and predict promoter activity in a gene-network context. However, we have just started utilizing these methods from Artificial Intelligence and it will probably take a few more years to achieve through them reliable in silico predictions of the performance in vivo of synthetic (and natural) promoters.

## Data Availability

Not applicable.

## References

[1] Elowitz M.B., Leibler S. (2000). A synthetic oscillatory network of transcriptional regulators. Nat. Cell Biol..

[2] Gardner T.S., Cantor C.R., Collins J.J. (2000). Construction of a genetic toggle switch in Escherichia coli. Nat. Cell Biol..

[3] Endy D. (2005). Foundations for engineering biology. Nat. Cell Biol..

[4] Pickens L.B., Tang Y., Chooi Y.-H. (2011). Metabolic Engineering for the Production of Natural Products. Annu. Rev. Chem. Biomol. Eng..

[5] Ro D.-K., Paradise E.M., Ouellet M., Fisher K.J., Newman K.L., Ndungu J.M., Ho K.A., Eachus R.A., Ham T.S., Kirby J. (2006). Production of the antimalarial drug precursor artemisinic acid in engineered yeast. Nat. Cell Biol..

[6] Liu Z., Zhang Y., Nielsen J. (2019). Synthetic Biology of Yeast. Biochem..

[7] Auxillos J.Y., Garcia-Ruiz E., Jones S., Li T., Jiang S., Dai J., Cai Y. (2019). Multiplex Genome Engineering for Optimizing Bioproduction in Saccharomyces cerevisiae. Biochem..

[8] Lu C., Jeffries T. (2007). Shuffling of Promoters for Multiple Genes to Optimize Xylose Fermentation in an Engineered Saccharomyces cerevisiae Strain. Appl. Environ. Microbiol..

[9] Wisselink H.W., Toirkens M.J., Berriel M.D.R.F., Winkler A.A., Van Dijken J.P., Pronk J.T., Van Maris A.J.A. (2007). Engineering of Saccharomyces cerevisiae for Efficient Anaerobic Alcoholic Fermentation of l-Arabinose. Appl. Environ. Microbiol..

[10] Da Silva N.A., Srikrishnan S. (2012). Introduction and expression of genes for metabolic engineering applications in Saccharomyces cerevisiae. FEMS Yeast Res..

[11] Nevoigt E., Kohnke J., Fischer C.R., Alper H., Stahl U., Stephanopoulos G. (2006). Engineering of Promoter Replacement Cassettes for Fine-Tuning of Gene Expression in Saccharomyces cerevisiae. Appl. Environ. Microbiol..

[12] Sun J., Shao Z., Zhao H., Nair N., Wen F., Xu J.-H., Zhao H. (2012). Cloning and characterization of a panel of constitutive promoters for applications in pathway engineering in Saccharomyces cerevisiae. Biotechnol. Bioeng..

[13] Redden H., Morse N., Alper H.S. (2014). The synthetic biology toolbox for tuning gene expression in yeast. FEMS Yeast Res..

[14] De Boer C.G., Vaishnav E.D., Sadeh R., Abeyta E.L., Friedman N., Regev A. (2020). Deciphering eukaryotic gene-regulatory logic with 100 million random promoters. Nat. Biotechnol..

[15] Hubmann G., Thevelein J.M., Nevoigt E. (2014). Natural and Modified Promoters for Tailored Metabolic Engineering of the Yeast Saccharomyces cerevisiae. Methods Mol. Biol..

[16] Hahn S., Young E.T. (2011). Transcriptional Regulation in Saccharomyces cerevisiae: Transcription Factor Regulation and Function, Mechanisms of Initiation, and Roles of Activators and Coactivators. Genet..

[17] Forsburg S.L., Guarente L. (1988). Mutational analysis of upstream activation sequence 2 of the CYC1 gene of Saccharomyces cerevisiae: A HAP2-HAP3-responsive site. Mol. Cell. Biol..

[18] Ogden J.E., Stanway C., Kim S., Mellor J., Kingsman A.J., Kingsman S.M. (1986). Efficient expression of the Saccharomyces cerevisiae PGK gene depends on an upstream activation sequence but does not require TATA sequences. Mol. Cell. Biol..

[19] Swamy K.B.S., Cho C.-Y., Chiang S., Tsai Z.T.-Y., Tsai H.-K. (2009). Impact of DNA-binding position variants on yeast gene expression. Nucleic Acids Res..

[20] Lubliner S., Keren L., Segal E. (2013). Sequence features of yeast and human core promoters that are predictive of maximal promoter activity. Nucleic Acids Res..

[21] Lubliner S., Regev I., Lotan-Pompan M., Edelheit S., Weinberger A., Segal E. (2015). Core promoter sequence in yeast is a major determinant of expression level. Genome Res..

[22] Redden H., Alper H.S. (2015). The development and characterization of synthetic minimal yeast promoters. Nat. Commun..

[23] Wobbe C.R., Struhl K. (1990). Yeast and human TATA-binding proteins have nearly identical DNA sequence requirements for transcription in vitro. Mol. Cell. Biol..

[24] Mishra A.K., Vanathi P., Bhargava P. (2003). The transcriptional activator GAL4-VP16 regulates the intra-molecular interactions of the TATA-binding protein. J. Biosci..

[25] Mogno I., Vallania F., Mitra R., Cohen B.A. (2010). TATA is a modular component of synthetic promoters. Genome Res..

[26] Zhang Z., Dietrich F.S. (2005). Mapping of transcription start sites in Saccharomyces cerevisiae using 5′ SAGE. Nucleic. Acids. Res..

[27] Hahn S., Hoar E.T., Guarente L. (1985). Each of three “TATA elements” specifies a subset of the transcription initiation sites at the CYC-1 promoter of Saccharomyces cerevisiae. Proc. Natl. Acad. Sci. USA.

[28] Guarente L., Lalonde B., Gifford P., Alani E. (1984). Distinctly regulated tandem upstream activation sites mediate catabolite repression of the CYC1 gene of S. cerevisiae. Cell.

[29] Guarente L., Ptashne M. (1981). Fusion of Escherichia coli lacZ to the cytochrome c gene of Saccharomyces cerevisiae. Proc. Natl. Acad. Sci. USA.

[30] Martens C., Krett B., Laybourn P.J. (2001). RNA polymerase II and TBP occupy the repressed CYC1 promoter. Mol. Microbiol..

[31] Guarente L., Yocum R.R., Gifford P. (1982). A GAL10-CYC1 hybrid yeast promoter identifies the GAL4 regulatory region as an upstream site. Proc. Natl. Acad. Sci. USA.

[32] Blazeck J., Liu L., Redden H., Alper H. (2011). Tuning Gene Expression in Yarrowia lipolytica by a Hybrid Promoter Approach. Appl. Environ. Microbiol..

[33] Guarente L. (1983). Yeast promoters and lacZ fusions designed to study expression of cloned genes in yeast. Methods Enzymol..

[34] Da Silva N.A., Bailey J.E. (1991). Influence of plasmid origin and promoter strength in fermentations of recombinant yeast. Biotechnol. Bioeng..

[35] Hadiji-Abbes N., Borchani-Chabchoub I., Triki H., Ellouz R., Gargouri A., Mokdad-Gargouri R. (2009). Expression of HBsAg and preS2-S protein in different yeast based system: A comparative analysis. Protein Expr. Purif..

[36] Sharon E., Kalma Y., Sharp A., Raveh-Sadka T., Levo M., Zeevi D., Keren L., Yakhini Z., Weinberger A., Segal E. (2012). Inferring gene regulatory logic from high-throughput measurements of thousands of systematically designed promoters. Nat. Biotechnol..

[37] Rajkumar A.S., Dénervaud N., Maerkl S.J. (2013). Mapping the fine structure of a eukaryotic promoter input-output function. Nat. Genet..

[38] Feng X., Marchisio M.A. (2021). Novel *S. cerevisiae* Hybrid Synthetic Promoters Based on Foreign Core Promoter Sequences. Int. J. Mol. Sci..

[39] Blazeck J., Garg R., Reed B., Alper H.S. (2012). Controlling promoter strength and regulation in Saccharomyces cerevisiae using synthetic hybrid promoters. Biotechnol. Bioeng..

[40] Bitter G.A., Egan K.M. (1988). Expression of interferon-gamma from hybrid yeast GPD promoters containing upstream regulatory sequences from the GAL1-GAL10 intergenic region. Gene.

[41] Purvis I.J., Chotai D., Dykes C.W., Lubahn D.B., French F.S., Wilson E.M., Hobden A.N. (1991). An androgen-inducible expression system for Saccharomyces cerevisiae. Gene.

[42] Iraqui I., Vissers S., Andreé B., Urrestarazu A. (1999). Transcriptional Induction by Aromatic Amino Acids in Saccharomyces cerevisiae. Mol. Cell. Biol..

[43] Kim S., Lee K., Bae S.-J., Hahn J.-S. (2015). Promoters inducible by aromatic amino acids and gamma-aminobutyrate (GABA) for metabolic engineering applications in Saccharomyces cerevisiae. Appl. Microbiol. Biotechnol..

[44] Leavitt J.M., Tong A., Tong J., Pattie J., Alper H.S. (2016). Coordinated transcription factor and promoter engineering to establish strong expression elements in Saccharomyces cerevisiae. Biotechnol. J..

[45] Brandman O., Stewart-Ornstein J., Wong D., Larson A., Williams C.C., Li G.-W., Zhou S., King D., Shen P.S., Weibezahn J. (2012). A Ribosome-Bound Quality Control Complex Triggers Degradation of Nascent Peptides and Signals Translation Stress. Cell.

[46] Zhang J., Sonnenschein N., Pihl T., Pedersen K.R., Jensen M.K., Keasling J.D. (2016). Engineering an NADPH/NADP+Redox Biosensor in Yeast. ACS Synth. Biol..

[47] Rajkumar A.S., Liu G., Bergenholm D., Arsovska D., Kristensen M., Nielsen J., Jensen M.K., Keasling J. (2016). Engineering of synthetic, stress-responsive yeast promoters. Nucleic Acids Res..

[48] Rajkumar A.S., Özdemir E., Lis A.V., Schneider K., Qin J., Jensen M.K., Keasling J.D. (2019). Engineered Reversal of Function in Glycolytic Yeast Promoters. ACS Synth. Biol..

[49] Ruohonen L., Penttilä M., Keränen S. (1991). Optimization ofBacillus α-amylase production bySaccharomyces cerevisiae. Yeast.

[50] Ruohonen L., Aalto M.K., Keranen S. (1995). Modifications to the ADH1 promoter of Saccharomyces cerevisiae for efficient production of heterologous proteins. J. Biotechnol..

[51] Hoshida H., Kondo M., Kobayashi T., Yarimizu T., Akada R. (2016). 5’-UTR introns enhance protein expression in the yeast Saccharomyces cerevisiae. Appl. Microbiol. Biotechnol..

[52] Raveh-Sadka T., Levo M., Shabi U., Shany B., Keren L., Lotan-Pompan M., Zeevi D., Sharon E., Weinberger A., Segal E. (2012). Manipulating nucleosome disfavoring sequences allows fine-tune regulation of gene expression in yeast. Nat. Genet..

[53] Alper H., Fischer C., Nevoigt E., Stephanopoulos G. (2005). Tuning genetic control through promoter engineering. Proc. Natl. Acad. Sci. USA.

[54] Du J., Yuan Y., Si T., Lian J., Zhao H. (2012). Customized optimization of metabolic pathways by combinatorial transcriptional engineering. Nucleic Acids Res..

[55] Ingolia N.T., Murray A.W. (2007). Positive-Feedback Loops as a Flexible Biological Module. Curr. Biol..

[56] Williams T., Averesch N., Winter G., Plan M., Vickers C., Nielsen L., Krömer J. (2015). Quorum-sensing linked RNA interference for dynamic metabolic pathway control in Saccharomyces cerevisiae. Metab. Eng..

[57] Curran K.A., Crook N.C., Karim A.S., Gupta A.R., Wagman A.M., Alper H.S. (2014). Design of synthetic yeast promoters via tuning of nucleosome architecture. Nat. Commun..

[58] Xi L., Fondufe-Mittendorf Y., Xia L., Flatow J., Widom J., Wang J.-P. (2010). Predicting nucleosome positioning using a duration Hidden Markov Model. BMC Bioinform..

[59] Myburgh M.W., Rose S.H., Viljoen-Bloom M. (2020). Evaluating and engineering Saccharomyces cerevisiae promoters for increased amylase expression and bioethanol production from raw starch. FEMS Yeast Res..

[60] Shi S., Choi Y.W., Zhao H., Tan M.H., Ang E.L. (2017). Discovery and engineering of a 1-butanol biosensor in Saccharomyces cerevisiae. Bioresour. Technol..

[61] Decoene T., De Maeseneire S.L., De Mey M. (2019). Modulating transcription through development of semi-synthetic yeast core promoters. PLoS ONE.

[62] Song W., Li J., Liang Q., Marchisio M.A. (2016). Can terminators be used as insulators into yeast synthetic gene circuits?. J. Biol. Eng..

[63] Guo Z., Sherman F. (1996). Signals sufficient for 3′-end formation of yeast mRNA. Mol. Cell. Biol..

[64] Ferrari G., Lamantea E., Donati A., Filosto M., Briem E., Carrara F., Parini R., Simonati A., Santer R., Zeviani M. (2005). Infantile hepatocerebral syndromes associated with mutations in the mitochondrial DNA polymerase-gammaA. Brain.

[65] Brambilla A., Mainieri D., Carbone M.L.A. (1997). A simple signal element mediates transcription termination and mRNA 3′ end formation in the DEG1 gene of Saccharomyces cerevisiae. Mol. Genet. Genom..

[66] Wertman K.F., Mount D.W. (1985). Nucleotide sequence binding specificity of the LexA repressor of Escherichia coli K-12. J. Bacteriol..

[67] Brent R., Ptashne M. (1981). Mechanism of action of the lexA gene product. Proc. Natl. Acad. Sci. USA.

[68] Brent R., Ptashne M. (1984). A bacterial repressor protein or a yeast transcriptional terminator can block upstream activation of a yeast gene. Nat. Cell Biol..

[69] Brent R., Ptashne M. (1985). A eukaryotic transcriptional activator bearing the DNA specificity of a prokaryotic repressor. Cell.

[70] Mizuno T., Wurtzel E.T., Inouye M. (1982). Osmoregulation of gene expression. II. DNA sequence of the envZ gene of the ompB operon of Escherichia coli and characterization of its gene product. J. Biol. Chem..

[71] Ruden D.M., Ma J., Li Y., Wood K., Ptashne M. (1991). Generating yeast transcriptional activators containing no yeast protein sequences. Nat. Cell Biol..

[72] Ma J., Ptashne M. (1987). A new class of yeast transcriptional activators. Cell.

[73] Keleher C.A., Redd M.J., Schultz J., Carlson M., Johnson A.D. (1992). Ssn6-Tup1 is a general repressor of transcription in yeast. Cell.

[74] Louvion J.-F., Havaux-Copf B., Picard D. (1993). Fusion of GAL4-VP16 to a steroid-binding domain provides a tool for gratuitous induction of galactose-responsive genes in yeast. Gene.

[75] Triezenberg S.J., Kingsbury R.C., McKnight S.L. (1988). Functional dissection of VP16, the trans-activator of herpes simplex virus immediate early gene expression. Genes Dev..

[76] McIsaac R.S., Silverman S.J., McClean M.N., Gibney P.A., Macinskas J., Hickman M., Petti A.A., Botstein D. (2011). Fast-acting and nearly gratuitous induction of gene expression and protein depletion inSaccharomyces cerevisiae. Mol. Biol. Cell.

[77] Gericke A., Hühnerfuss H. (1994). IR reflection absorption spectroscopy: A versatile tool for studying interfacial enzymatic processes. Chem. Phys. Lipids.

[78] Ajo-Franklin C.M., Drubin D.A., Eskin J.A., Gee E.P., Landgraf D., Phillips I., Silver P.A. (2007). Rational design of memory in eukaryotic cells. Genes Dev..

[79] Ottoz D.S., Rudolf F., Stelling J. (2014). Inducible, tightly regulated and growth condition-independent transcription factor in Saccharomyces cerevisiae. Nucleic Acids Res..

[80] Dossani Z.Y., Apel A.R., Szmidt-Middleton H., Hillson N.J., Deutsch S., Keasling J.D., Mukhopadhyay A. (2017). A combinatorial approach to synthetic transcription factor-promoter combinations for yeast strain engineering. Yeast.

[81] Rantasalo A., Czeizler E., Virtanen R., Rousu J., Lähdesmäki H., Penttilä M., Jäntti J., Mojzita D. (2016). Synthetic Transcription Amplifier System for Orthogonal Control of Gene Expression in Saccharomyces cerevisiae. PLoS ONE.

[82] Rantasalo A., Kuivanen J., Penttilä M., Jäntti J., Mojzita D. (2018). Synthetic Toolkit for Complex Genetic Circuit Engineering in Saccharomyces cerevisiae. ACS Synth. Biol..

[83] Labow M.A., Baim S.B., Shenk T., Levine A.J. (1990). Conversion of the lac repressor into an allosterically regulated transcriptional activator for mammalian cells. Mol. Cell. Biol..

[84] Rantasalo A., Landowski C.P., Kuivanen J., Korppoo A., Reuter L., Koivistoinen O., Valkonen M., Penttilä M., Jäntti J., Mojzita D. (2018). A universal gene expression system for fungi. Nucleic Acids Res..

[85] Hillen W., Berens C. (1994). Mechanisms Underlying Expression of TN10 Encoded Tetracycline Resistance. Annu. Rev. Microbiol..

[86] Gossen M., Bujard H. (1992). Tight control of gene expression in mammalian cells by tetracycline-responsive promoters. Proc. Natl. Acad. Sci. USA.

[87] Gossen M., Freundlieb S., Bender G., Muller G., Hillen W., Bujard H. (1995). Transcriptional activation by tetracyclines in mammalian cells. Science.

[88] Garí E., Piedrafita L., Aldea M., Herrero E. (1997). A Set of Vectors with a Tetracycline-Regulatable Promoter System for Modulated Gene Expression inSaccharomyces cerevisiae. Yeast.

[89] Belli G. (1998). An activator/repressor dual system allows tight tetracycline-regulated gene expression in budding yeast. Nucleic Acids Res..

[90] Cuperus J.T., Lo R.S., Shumaker L., Proctor J., Fields S. (2015). A tetO Toolkit to Alter Expression of Genes in Saccharomyces cerevisiae. ACS Synth. Biol..

[91] Mnaimneh S., Davierwala A.P., Haynes J., Moffat J., Peng W.-T., Zhang W., Yang X., Pootoolal J., Chua G., Lopez A. (2004). Exploration of Essential Gene Functions via Titratable Promoter Alleles. Cell.

[92] Murphy K.F., Balázsi G., Collins J.J. (2007). Combinatorial promoter design for engineering noisy gene expression. Proc. Natl. Acad. Sci. USA.

[93] Ellis T., Wang X., Collins J.J. (2009). Diversity-based, model-guided construction of synthetic gene networks with predicted functions. Nat. Biotechnol..

[94] Blount B.A., Weenink T., Vasylechko S., Ellis T. (2012). Rational Diversification of a Promoter Providing Fine-Tuned Expression and Orthogonal Regulation for Synthetic Biology. PLoS ONE.

[95] Oehler S., Amouyal M., Kolkhof P., von Wilcken-Bergmann B., Müller-Hill B. (1994). Quality and position of the three lac operators of *E. coli* define efficiency of repression. EMBO J..

[96] Grilly C., Stricker J., Pang W.L., Bennett M.R., Hasty J. (2007). A synthetic gene network for tuning protein degradation in Saccharomyces cerevisiae. Mol. Syst. Biol..

[97] Marchisio M.A. (2014). In silico design and in vivo implementation of yeast gene Boolean gates. J. Biol. Eng..

[98] Mazumder M., McMillen D.R. (2014). Design and characterization of a dual-mode promoter with activation and repression capability for tuning gene expression in yeast. Nucleic Acids Res..

[99] Gnugge R., Dharmarajan L., Lang M., Stelling J. (2016). An Orthogonal Permease-Inducer-Repressor Feedback Loop Shows Bistability. ACS Synth. Biol..

[100] Bandiera L., Hou Z., Kothamachu V.B., Balsa-Canto E., Swain P.S., Menolascina F. (2018). On-Line Optimal Input Design Increases the Efficiency and Accuracy of the Modelling of an Inducible Synthetic Promoter. Processes.

[101] Rodriguez G.M., Hussain M.S., Gambill L., Gao D., Yaguchi A., Blenner M. (2016). Engineering xylose utilization in Yarrowia lipolytica by understanding its cryptic xylose pathway. Biotechnol. Biofuels.

[102] Teo W.S., Chang M.W. (2015). Bacterial XylRs and synthetic promoters function as genetically encoded xylose biosensors inSaccharomyces cerevisiae. Biotechnol. J..

[103] Wang M., Li S., Zhao H. (2016). Design and engineering of intracellular-metabolite-sensing/regulation gene circuits inSaccharomyces cerevisiae. Biotechnol. Bioeng..

[104] Hector R.E., Mertens J.A. (2017). A Synthetic Hybrid Promoter for Xylose-Regulated Control of Gene Expression in Saccharomyces Yeasts. Mol. Biotechnol..

[105] Wei W.-P., Shang Y., Zhang P., Liu Y., You D., Yin B.-C., Ye B.-C. (2020). Engineering Prokaryotic Transcriptional Activator XylR as a Xylose-Inducible Biosensor for Transcription Activation in Yeast. ACS Synth. Biol..

[106] Teo W.S., Hee K.S., Chang M.W. (2013). Bacterial FadR and synthetic promoters function as modular fatty acid sensor- regulators inSaccharomyces cerevisiae. Eng. Life Sci..

[107] Teo W.S., Chang M.W. (2014). Development and characterization of AND-gate dynamic controllers with a modular synthetic GAL1 core promoter inSaccharomyces cerevisiae. Biotechnol. Bioeng..

[108] Li S., Si T., Wang M., Zhao H. (2015). Development of a Synthetic Malonyl-CoA Sensor in Saccharomyces cerevisiae for Intracellular Metabolite Monitoring and Genetic Screening. ACS Synth. Biol..

[109] David F., Nielsen J., Siewers V. (2016). Flux Control at the Malonyl-CoA Node through Hierarchical Dynamic Pathway Regulation in Saccharomyces cerevisiae. ACS Synth. Biol..

[110] Dabirian Y., Teixeira P.G., Nielsen J., Siewers V., David F. (2019). FadR-Based Biosensor-Assisted Screening for Genes Enhancing Fatty Acyl-CoA Pools in Saccharomyces cerevisiae. ACS Synth. Biol..

[111] Dabirian Y., Li X., Chen Y., David F., Nielsen J., Siewers V. (2019). Expanding the Dynamic Range of a Transcription Factor-Based Biosensor in Saccharomyces cerevisiae. ACS Synth. Biol..

[112] Ambri F., D’Ambrosio V., Di Blasi R., Maury J., Jacobsen S.A.B., McCloskey D., Jensen M.K., Keasling J.D. (2019). High-Resolution Scanning of Optimal Biosensor Reporter Promoters in Yeast. ACS Synth. Biol..

[113] Qiu C., Chen X., Rexida R., Shen Y., Qi Q., Bao X., Hou J. (2020). Engineering transcription factor-based biosensors for repressive regulation through transcriptional deactivation design in Saccharomyces cerevisiae. Microb. Cell Factories.

[114] Umeyama T., Okada S., Ito T. (2013). Synthetic Gene Circuit-Mediated Monitoring of Endogenous Metabolites: Identification ofGAL11as a Novel Multicopy Enhancer ofS-Adenosylmethionine Level in Yeast. ACS Synth. Biol..

[115] Skjoedt M.L., Snoek T., Kildegaard K.R., Arsovska D., Eichenberger M., Goedecke T.J., Rajkumar A.S., Zhang J., Kristensen M., Lehka B.J. (2016). Engineering prokaryotic transcriptional activators as metabolite biosensors in yeast. Nat. Chem. Biol..

[116] Mandell J.G., Barbas C.F. (2006). Zinc Finger Tools: Custom DNA-binding domains for transcription factors and nucleases. Nucleic Acids Res..

[117] Khalil A.S., Lu T.K., Bashor C.J., Ramirez C.L., Pyenson N.C., Joung J.K., Collins J.J. (2012). A Synthetic Biology Framework for Programming Eukaryotic Transcription Functions. Cell.

[118] McIsaac R.S., Oakes B.L., Wang X., Dummit K.A., Botstein D., Noyes M.B. (2012). Synthetic gene expression perturbation systems with rapid, tunable, single-gene specificity in yeast. Nucleic Acids Res..

[119] McIsaac R.S., Gibney P.A., Chandran S.S., Benjamin K.R., Botstein D. (2014). Synthetic biology tools for programming gene expression without nutritional perturbations in Saccharomyces cerevisiae. Nucleic Acids Res..

[120] Kotopka B.J., Smolke C.D. (2020). Model-driven generation of artificial yeast promoters. Nat. Commun..

[121] Bogdanove A.J., Voytas D. (2011). TAL Effectors: Customizable Proteins for DNA Targeting. Sci..

[122] De Lange O., Wolf C., Thiel P., Krüger J., Kleusch C., Kohlbacher O., Lahaye T. (2015). DNA-binding proteins from marine bacteria expand the known sequence diversity of TALE-like repeats. Nucleic. Acids. Res..

[123] Machens F., Balazadeh S., Mueller-Roeber B., Messerschmidt K. (2017). Synthetic Promoters and Transcription Factors for Heterologous Protein Expression in Saccharomyces cerevisiae. Front. Bioeng. Biotechnol..

[124] Ishino Y., Shinagawa H., Makino K., Amemura M., Nakata A. (1987). Nucleotide sequence of the iap gene, responsible for alkaline phosphatase isozyme conversion in Escherichia coli, and identification of the gene product. J. Bacteriol..

[125] Horvath P., Barrangou R. (2010). CRISPR/Cas, the immune system of bacteria and archaea. Science.

[126] Marchisio M.A., Huang Z. (2017). CRISPR-Cas type II-based Synthetic Biology applications in eukaryotic cells. RNA Biol..

[127] Jinek M., Chylinski K., Fonfara I., Hauer M., Doudna J.A., Charpentier E. (2012). A Programmable dual-RNA-guided DNA endonuclease in adaptive bacterial immunity. Science.

[128] Qi L.S., Larson M.H., Gilbert L.A., Doudna J.A., Weissman J.S., Arkin A.P., Lim W.A. (2013). Repurposing CRISPR as an RNA-Guided Platform for Sequence-Specific Control of Gene Expression. Cell.

[129] Chavez A., Tuttle M., Pruitt B.W., Ewen-Campen B., Chari R., Ter-Ovanesyan D., Haque S.J., Cecchi R.J., Kowal E.J.K., Buchthal J. (2016). Comparison of Cas9 activators in multiple species. Nat. Methods.

[130] Farzadfard F., Perli S.D., Lu T.K. (2013). Tunable and Multifunctional Eukaryotic Transcription Factors Based on CRISPR/Cas. ACS Synth. Biol..

[131] Gilbert L.A., Larson M.H., Morsut L., Liu Z., Brar G.A., Torres S.E., Stern-Ginossar N., Brandman O., Whitehead E.H., Doudna J.A. (2013). CRISPR-Mediated Modular RNA-Guided Regulation of Transcription in Eukaryotes. Cell.

[132] Zalatan J.G., Lee M.E., Almeida R., Gilbert L.A., Whitehead E.H., La Russa M., Tsai J., Weissman J.S., Dueber J.E., Qi L.S. (2015). Engineering Complex Synthetic Transcriptional Programs with CRISPR RNA Scaffolds. Cell.

[133] Gander M.W., Vrana J.D., Voje W.E., Carothers J.M., Klavins E. (2017). Digital logic circuits in yeast with CRISPR-dCas9 NOR gates. Nat. Commun..

[134] Gao Y., Zhao Y. (2014). Self-processing of ribozyme-flanked RNAs into guide RNAs in vitro and in vivo for CRISPR-mediated genome editing. J. Integr. Plant Biol..

[135] Alon U. (2006). An Introduction to Systems Biology: Design Principles of Biological Circuits.

[136] Klipp E., Liebermeister W., Wierling C., Kowald A., Lehrach H., Herwig R. (2009). Systems Biology: A Textbook.

